# Evaluation of Wideband Pulse Sequences for Suppressing Image Artifacts in Children with Cardiac Implantable Electronic Devices

**DOI:** 10.1148/ryct.250310

**Published:** 2025-11-06

**Authors:** Oluyemi Aboyewa, Andrada Popescu, Simon Lee, Joseph Camarda, KyungPyo Hong, Dhaivat Shah, Christina Laternser, Laleh Golestanirad, Daniel Kim, Gregory Webster

**Affiliations:** ^1^Department of Biomedical Engineering, McCormick School of Engineering, Northwestern University, Evanston, Ill; ^2^Department of Radiology, Feinberg School of Medicine, Northwestern University, 737 N Michigan Ave, Ste 1600, Chicago, IL 60611; ^3^Department of Medical Imaging, Ann & Robert H. Lurie Children’s Hospital, Chicago, Ill; ^4^Division of Cardiology, Department of Pediatrics, Ann & Robert H. Lurie Children’s Hospital, Chicago, Ill

**Keywords:** Wideband, Pediatric, Late Gadolinium Enhancement, Perfusion, Pacemaker, Defibrillator

## Abstract

**Purpose:**

To determine whether wideband late gadolinium enhancement (LGE) and wideband perfusion pulse sequences suppress image artifacts in children in the presence of a cardiac implantable electronic device (CIED).

**Materials and Methods:**

In the first experiment of this prospective study, unenhanced cardiac MRI was performed at 1.5 T in 18 healthy pediatric participants (median age, 12 years [range, 10–15 years]; 12 [67%] female) with and without a CIED generator taped in the subclavicular and abdominal positions. LGE and perfusion scans were performed with standard and wideband sequences in each state. Three clinical raters adjudicated the presence or absence of clinically relevant image artifacts. Image artifacts were also quantified. In the second experiment, wideband perfusion and LGE MRI scans with gadolinium-based contrast material administration were performed on four pediatric participants (median age, 15 years [range, 15–19 years]; three [75%] female) with CIEDs in situ. Image quality was graded on a five-point Likert scale by clinical raters (3 = clinically acceptable). Myocardial blood flow was also quantified.

**Results:**

In the participant experiments, standard LGE produced image artifacts in 61.81% (178 of 288) and 37.15% (107 of 288) of myocardial segments for subclavicular and abdominal IPGs, respectively, and were significantly different (*P* < .001) from wideband LGE (14.68% [42 of 288] and 0%, respectively) for both positions. Quantitative analysis results were consistent with expert adjudication. Wideband cardiac MRI in four children with CIEDs achieved high image quality (median score ≥ 4.0) and mean resting myocardial blood flow value of 1.06 mL/min/g (*n* = 3).

**Conclusion:**

Compared with standard LGE and perfusion MRI, wideband LGE and perfusion MRI produced significantly fewer image artifacts induced by CIEDs in pediatric participants.

**Keywords:** Wideband, Pediatric, Late Gadolinium Enhancement, Perfusion, Pacemaker, Defibrillator

*Supplemental material is available for this article.*

© The Author(s) 2025. Published by the Radiological Society of North America under a CC BY 4.0 license.

**Summary**

This study compared the diagnostic yield of wideband late gadolinium enhancement and perfusion pulse sequences to their standard cardiovascular MRI counterparts in pediatric participants using an implantable pulse generator taped at anatomically correct locations to mimic both endocardial and epicardial cardiac implantable electronic device systems.

**Key Points**

■ Wideband sequences suppressed image artifacts due to the implantable pulse generator, increasing the number of myocardial segments that could be interpreted clinically in the participant study.
■ Wideband sequences produced high diagnostic image quality in pediatric participants with cardiac implantable electronic devices in situ.

## Introduction

Cardiovascular MRI is routinely used to diagnose and monitor patients with congenital or acquired heart diseases (1,2). However, the utility of cardiovascular MRI in children with cardiac implantable electronic devices (CIEDs) is less proven. Most CIEDs consist of an implantable pulse generator (IPG) and leads that connect to the heart. However, the presence of an IPG creates local inhomogeneity in the magnetic field of the MRI system, causing highly off-resonant effects. These effects induce signal loss (ie, intravoxel spin dephasing) and hyperintense artifacts, resulting in a low diagnostic yield of cardiovascular MRI (3,4).

To address this, wideband late gadolinium-enhanced (LGE) (5,6), perfusion (7,8), and T1 mapping (9) sequences have been developed. In wideband sequences, inversion or saturation recovery pulses are designed with a higher spectral bandwidth than conventional pulses, sufficient to excite off-resonant spins and suppress hyperintense artifacts due to the IPG (5,7,9,10).

Although the clinical utility of wideband sequences has been shown in adult patients with endocardial CIEDs (5,8,11–13), reports in children are nearly nonexistent. An additional challenge in pediatric patients is the size of the IPG relative to the body size and the proximity of the IPG to the heart (14). It is, therefore, clinically relevant to quantify the sensitivity of cardiovascular MRI sequences to IPGs in a pediatric context. The purpose of this study was to compare the diagnostic yield of wideband LGE and perfusion pulse sequences to their standard counterparts in a controlled experiment of pediatric participants using an IPG taped at anatomically correct locations to mimic both endocardial and epicardial CIED systems. We also reported initial wideband cardiovascular MRI results from a small cohort of pediatric participants with CIED systems in situ.

## Materials and Methods

### Experiment in Healthy Participants

Healthy participants between 9 and 18 years of age were prospectively recruited (Table 1). Children were excluded if they had intrathoracic pathology, had any contraindication to MRI, had allergies to any study materials, could not communicate adequately with the MRI technical staff, or had a skin condition on the chest. The study protocol was approved by the Lurie Children’s Hospital of Chicago institutional review board. Written informed consent was obtained from the participants’ parents.

**Table 1:** Participant Demographics and Body Size

Participant Characteristic	No. of Healthy Participants (*n* = 18)	No. of Participants (*n* = 4)
Age (y)	12 (10–15)	15 (15–19)
Weight (kg)	50.2 (37.5–95.4)	73.5 (50.5–95.0)
Height (cm)	157.3 (139.5–183.5)	171.0 (141.0–181.0)
Body mass index	20.4 (18.0–28.7)	25.7 (23.5–28.9)
Body surface area (m^2^)	1.5 (1.2–2.2)	1.9 (1.4–2.2)
Sex		
Female	12 (67)	3 (75)
Left ventricular ejection fraction (%)	60.4 ± 4.4	50.5 ± 16.4
Diagnosis		
Atrioventricular block	…	1
Anthracycline cardiotoxicity	…	1
Ventricular tachycardia	…	1
Hypertrophic cardiomyopathy	…	1
Device		
Pacemaker		
ADSR01 Adapta; Medtronic	…	1
A2DR01 Advisa DR; Medtronic	…	1
Implantable cardioverter defibrillator		
DVFB1D4 Visia AF MRI VR; Medtronic	…	2

Note.—Data are reported as medians with ranges in parentheses, numbers with percentages in parentheses, or means ± SDs. Body mass index is calculated as weight in kilograms divided by height in meters squared.

Participants were oriented to the MRI scanner, and cardiac rhythm monitoring was placed. To simulate maximal artifact (ie, a worst-case scenario), we used a large implantable cardioverter defibrillator pulse generator (Evera; Medtronic). Implantation was simulated by wrapping the IPG in cloth to minimize potential heating from the skin-metal contact. No leads were affixed to the IPG.

**Figure 1:**
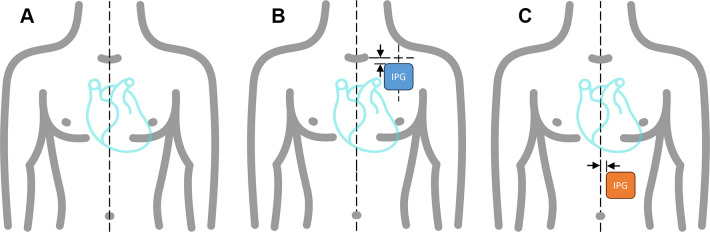
Schematic of the implantable pulse generator (IPG) simulation in the three experimental states for cardiovascular MRI acquisition **(A)** without an IPG, **(B)** with an IPG taped to the skin 1 cm below the left clavicle with the medial device edge at the midclavicular line (simulating endocardial IPG), and **(C)** with an IPG taped 1 cm left of the midabdominal line with the upper edge of the IPG 1 cm below the lowest anterior rib (simulating epicardial IPG). Standard and wideband late gadolinium enhancement as well as perfusion sequences were performed in each state.

Each participant was scanned in three experimental states: no IPG, subclavicular IPG, and abdominal IPG (Fig 1). Subclavicular devices were taped 1 cm below the left clavicle with the medial device edge at the midclavicular line. Abdominal devices were taped 1 cm left of the midabdominal line with the upper edge of the IPG 1 cm below the lowest anterior rib.

**MRI and pulse sequence**

All cardiovascular MRI studies were performed on the same whole-body 1.5-T MRI scanner (Aera; Siemens Healthineers). To minimize exposure risks for pediatric participants, a contrast agent was not administered. In each experimental state, participants were scanned with four protocols: wideband LGE (6), wideband perfusion (8) and corresponding clinical standard, LGE, and perfusion sequences with nearly matched imaging parameters.

The LGE scans were performed with phase-sensitive inversion recovery reconstruction (15). Approximately 10–12 short-axis sections were scanned to cover the whole heart, with the inversion time set to null the native myocardium. For the perfusion scan, we acquired proton density and T1-weighted images in the basal, mid, and apical short-axis sections. For more details on the pulse sequence and imaging parameters, please see Appendix S1 and Table S1, respectively.

**Image quality assessment and quantitative analysis**

Three experienced cardiovascular MRI raters (A.P., radiologist with 13 years of experience; J.C., cardiologist with 12 years of experience; and S.L., cardiologist with 8 years of experience) adjudicated artifacts in the LGE images. For each scan, the basal, mid, and apical sections were identified; then the images were separated, encoded, and randomized. The raters first calibrated their scoring system in consensus on training datasets, then adjudicated each scan independently in the 16 American Heart Association left ventricular (LV) myocardium segments on a binary scale (16). A clinically relevant artifact was present if greater than 50% of the segment had a hyperintense artifact; otherwise, no relevant artifact was present. The majority score for each segment was retained for analysis. Adjudication was not performed for the perfusion images in the healthy pediatric participants because no gadolinium-based contrast agent was used, and there was no contrast between the myocardium and the blood pool.

We quantified the hyperintense artifact in both LGE and perfusion images in all experimental states. For both analyses, the standard and wideband scans with no taped IPG were used as references for their respective scan types. Please see Appendix S2 for details on the quantitative artifact analysis.

### Experiment in Participants with CIEDs in Situ

We included four pediatric participants (Table 1) with CIEDs in situ who were scanned clinically at Lurie Children’s Hospital of Chicago between 2021 and 2025. As per hospital policy, all pediatric participants or parents provided written consent, as cardiovascular MRI is considered an off-label use in pediatric participants with CIEDs. All participants had an endocardial CIED, including an IPG and a lead in place. All studies were performed on one 1.5-T MRI scanner (Aera; Siemens Healthineers). For perfusion imaging, an additional arterial input function short-axis plane was acquired to allow for myocardial blood flow quantification (17). A gadolinium-based contrast agent (gadobutrol, 0.075 mmol/kg) was administered intravenously at 3 mL/sec, followed by a 20-mL saline flush.

To evaluate the image quality in participants with CIEDs, the three clinical raters assigned visual scores for each LV segment on a five-point Likert scale, ranging from 1, nondiagnostic, to 5, excellent, with a score of 3 defined as clinically acceptable. In addition, an image artifact score was assigned for each segment on a binary scale that was the same as that of the healthy participant. Clinical adjudication was performed for both wideband LGE and perfusion images. For details on the myocardial blood flow quantification for the wideband perfusion scans, please see Appendix S3.

### Statistical Analysis

All analyses were done in MATLAB. For the expert adjudication of the LGE images in the healthy participants, we averaged the score for each segment for display purposes only. The overall artifact in each segment was assigned as either clinically interpretable or clinically uninterpretable, based on the majority review of the expert adjudications. We performed Fisher exact tests to compare the number of clinically uninterpretable segments, and *P* less than .05 was considered statistically significant.

For the quantitative artifact analysis in the LGE images of the healthy participant, the mean signal intensity (SI) of each myocardial segment was averaged across the participants. A segment had a hyperintense artifact if the average SI was greater than the mean SI + 3 SD of the reference. Using this artifact threshold, we constructed a binary table across segments and participants. We then performed Fisher exact tests to compare the rate of artifacts between IPG positions (ie, subclavicular vs abdominal IPG) and scan types (standard vs wideband). The same analyses were performed for the perfusion sequence across the three short-axis sections.

## Results

### Healthy Participants

**Figure 2:**
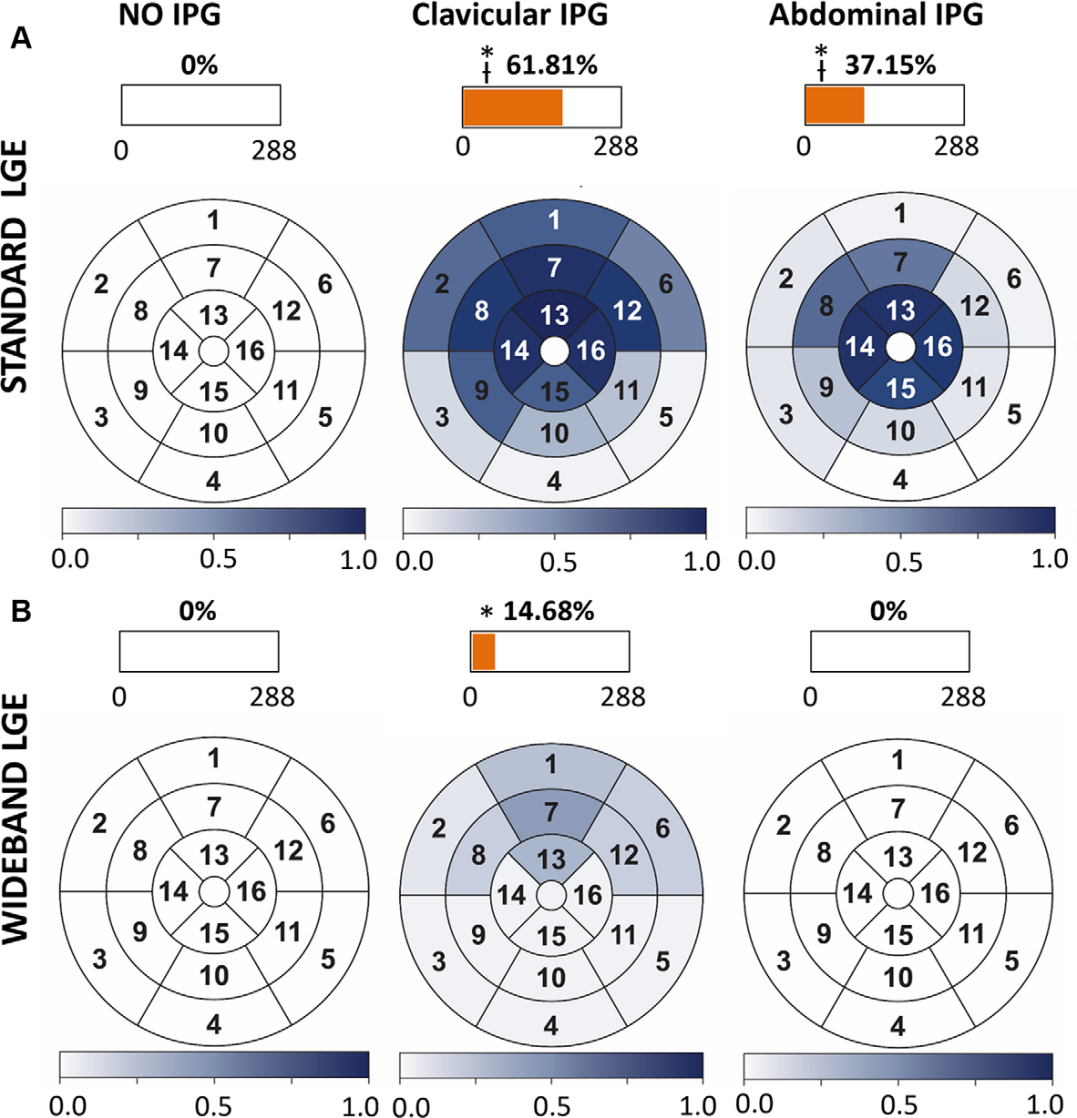
Expert adjudication of hyperintense artifact in **(A)** standard and **(B)** wideband late gadolinium enhancement (LGE) images of healthy participants, scanned in three experimental states; no implantable pulse generator (IPG) (left column), endocardial IPG (middle column), and epicardial IPG (right column) simulated with IPG taped to the subclavicular and abdominal position, respectively. The bull’s-eye plots show the rate of artifacts for each left ventricular myocardial segment, averaged across the participants (*n* = 18), for all scan types and IPG positions. The box plot shows the prevalence of artifacts (in percentage) of the total adjudicated segments for each scan type and IPG positions. *P < .001 compared with reference (no IPG). *P* < .001 compared between standard and wideband LGE with IPG.

LGE images were obtained from 18 healthy participants (median age, 12 years [range, 10–15 years]; 12 [67%] female) with a normal LV ejection fraction (60.4% ± 4.4 [SD]; Table 1). The expert raters adjudicated 288 segments for each scan type and experimental state. There were no clinically significant artifacts (0 of 288 segments) in the standard and wideband LGE with no IPG. However, in standard LGE with IPG taped in the subclavicular and abdominal positions, 61.81% (178 of 288) and 37.15% (107 of 288) of the segments (Fig 2A) were clinically uninterpretable, respectively, and were significantly different from the reference (*P* < .001). In contrast, when wideband LGE sequences were used, fewer segments, 14.68% (42 of 288) and 0% (Fig 2B), were clinically uninterpretable with an IPG taped in the subclavicular and abdominal position, respectively. Only wideband LGE in the subclavicular position was significantly different (*P* < .001) from its reference. Nevertheless, the number of uninterpretable segments was significantly different (*P* < .001) between standard and wideband LGE for both positions. Figure 2 also shows bull’s-eye plots of the rate of artifacts for each segment, averaged across the participants, for all scan types and IPG positions.

**Figure 3:**
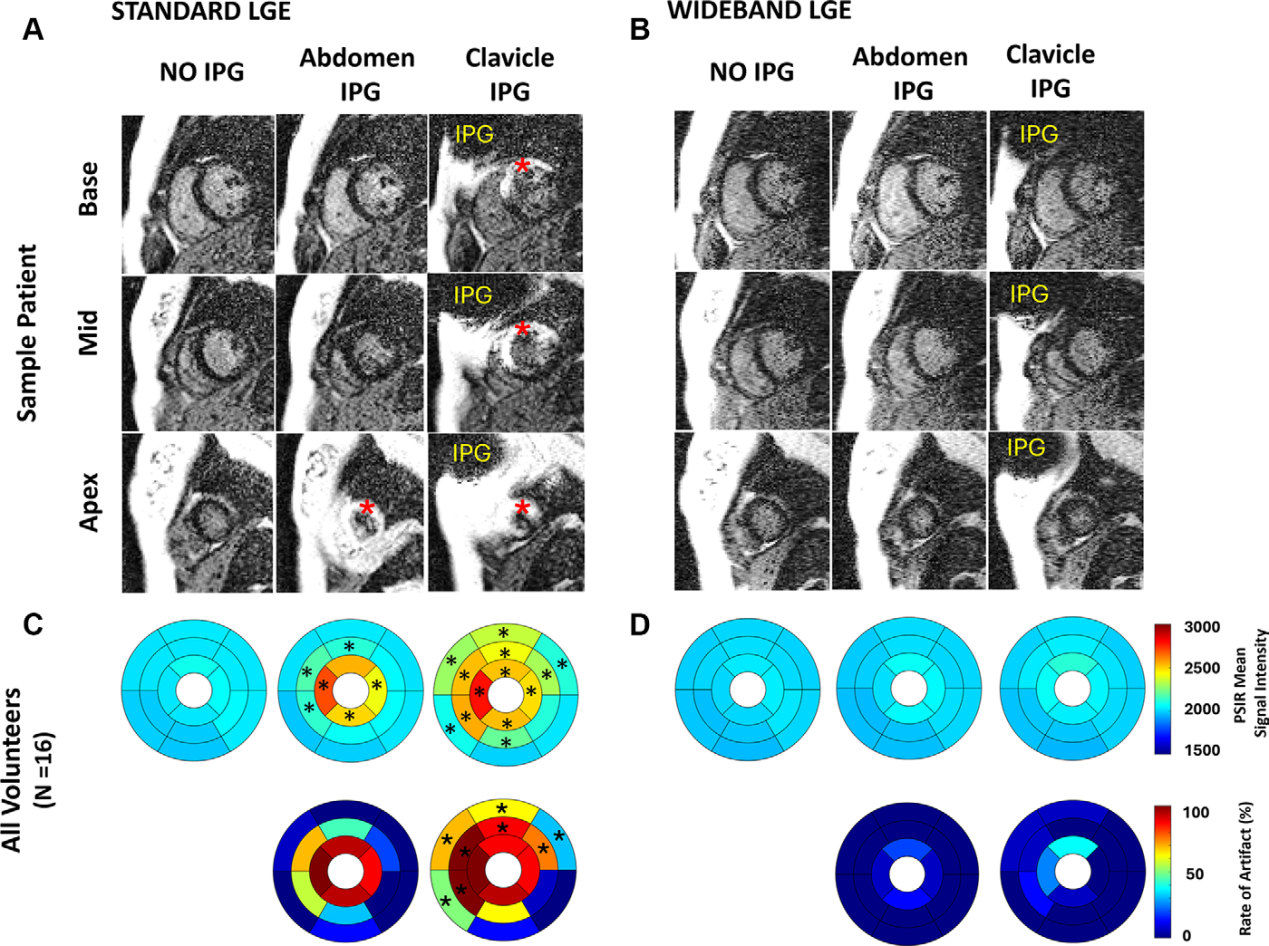
Sample images of the three short-axis sections (base, mid, and apex) of the unenhanced **(A)** standard and **(B)** wideband late gadolinium enhancement (LGE) with and without the implantable pulse generator (IPG) taped to the subclavicular and abdominal position of the healthy participant. The red asterisks indicate hyperintense artifacts. The mean signal intensity of the phase-sensitive inversion recovery images (*n* = 16) for **(C)** standard and **(D)** wideband is shown in the bull’s-eye plots (top row). Myocardial segments with an asterisk fall outside the mean signal intensity + 3 SD (artifact threshold) of that of the standard and wideband LGE without IPG (reference). In addition, the rate of artifacts in each myocardial segment is shown in the bull’s-eye plots (bottom row); segments with an asterisk are significantly different (*P* < .04) for both IPG positions. PSIR = phase-sensitive inversion recovery.

For the quantitative artifact analysis in the LGE images, we excluded two participants because phase-sensitive inversion recovery reconstruction was not included by mistake. Averaging the results over 16 participants, the standard LGE produced significant hyperintense artifacts in 13 of 16 and seven of 16 LV segments with an IPG taped in the subclavicular and abdominal positions, respectively, compared with the reference with no IPG (Fig 3A). The rate of artifacts was significantly different (*P* < .04) in eight of 16 segments between the two taped IPG positions. In contrast, wideband LGE effectively suppressed the hyperintense artifacts across all myocardial segments and was not significantly different between both taped positions (*P* > .33, Fig 3B). The number of segments with artifacts was significantly different (*P* < .01) between standard and wideband LGE for both positions.

**Figure 4:**
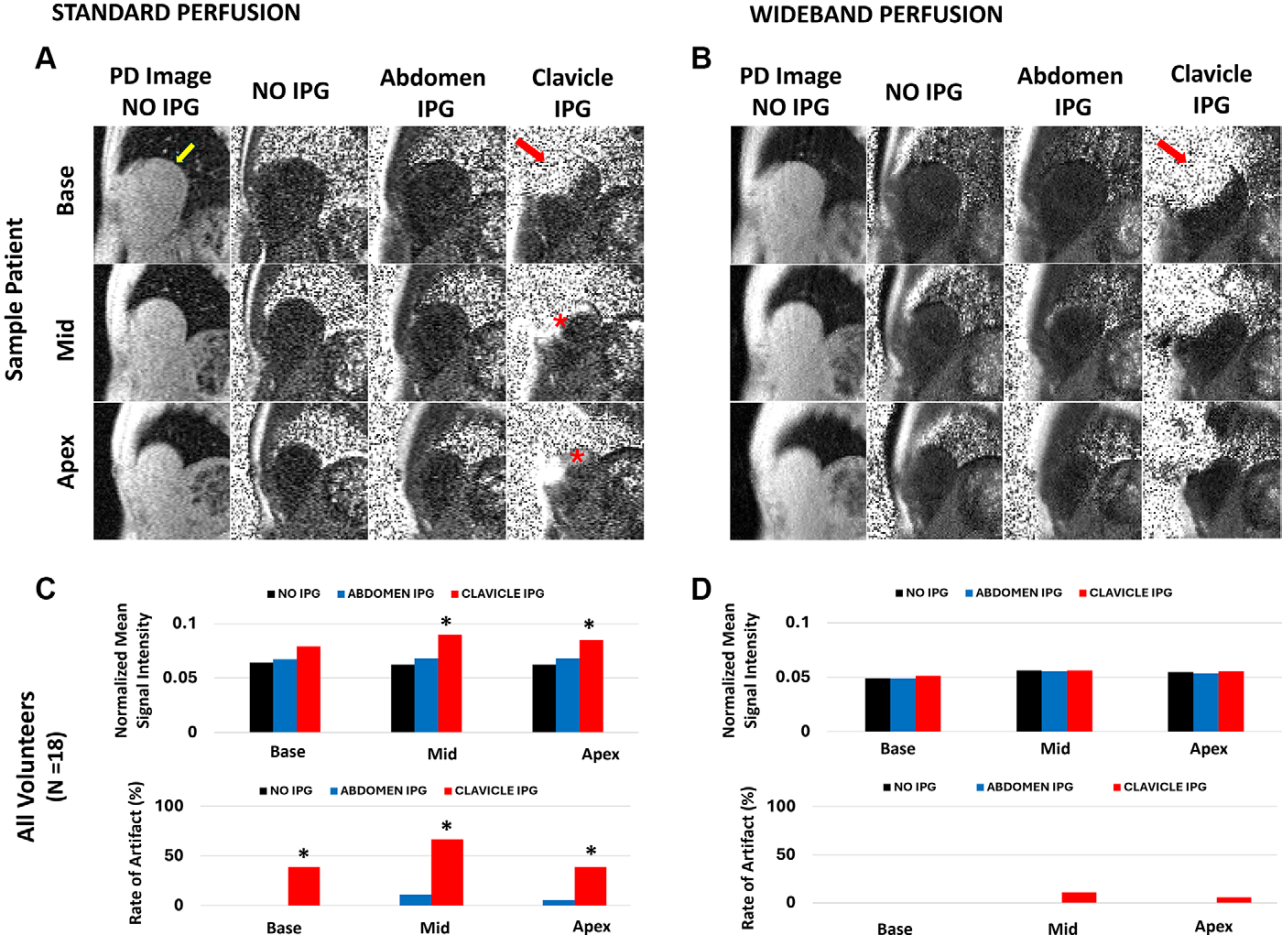
Sample proton density (PD) and normalized images of the three short-axis sections (base, mid, and apex) of the unenhanced **(A)** standard and **(B)** wideband perfusion with and without the implantable pulse generator (IPG) taped to the subclavicular and abdominal position of the healthy participants. The yellow arrow indicates the heart, while the red arrow indicates the signal void caused by the IPG in the normalized image. The red asterisks indicate hyperintense artifacts. The normalized mean signal intensity (*n* = 18) of the whole left and right ventricles of the three sections for **(C)** standard and **(D)** wideband is shown in the bar chart (top row). Sections with asterisks fall outside of the mean signal intensity + 3 SD (artifact threshold) of the standard and wideband without the IPG (reference). We specifically excluded voxels with signal voids to avoid spurious values arising from division by zero during image normalization. In addition, the rate of artifacts in each myocardial section is shown in the bar chart (bottom row); sections with an asterisk are significantly different (*P* < .04) for both IPG positions.

Figure 4 compares the diagnostic yield of standard and wideband perfusion sequences in one representative participant and summarizes results from all 18 participants. Standard perfusion produced significant hyperintense artifacts in two of the three short-axis planes (mid and apical), whereas wideband perfusion suppressed these artifacts in all sections. The artifact rate with standard perfusion differed significantly (*P* < .04) between the two taped IPG positions in all three sections. In contrast, wideband LGE effectively suppressed hyperintense artifacts across all sections, with a nonsignificant (*P* > .49) difference between IPG positions. Image artifacts were significantly different (*P* < .04) between standard and wideband perfusion when the IPG was taped in the subclavicular position.

### Participants with CIEDs

**Figure 5:**
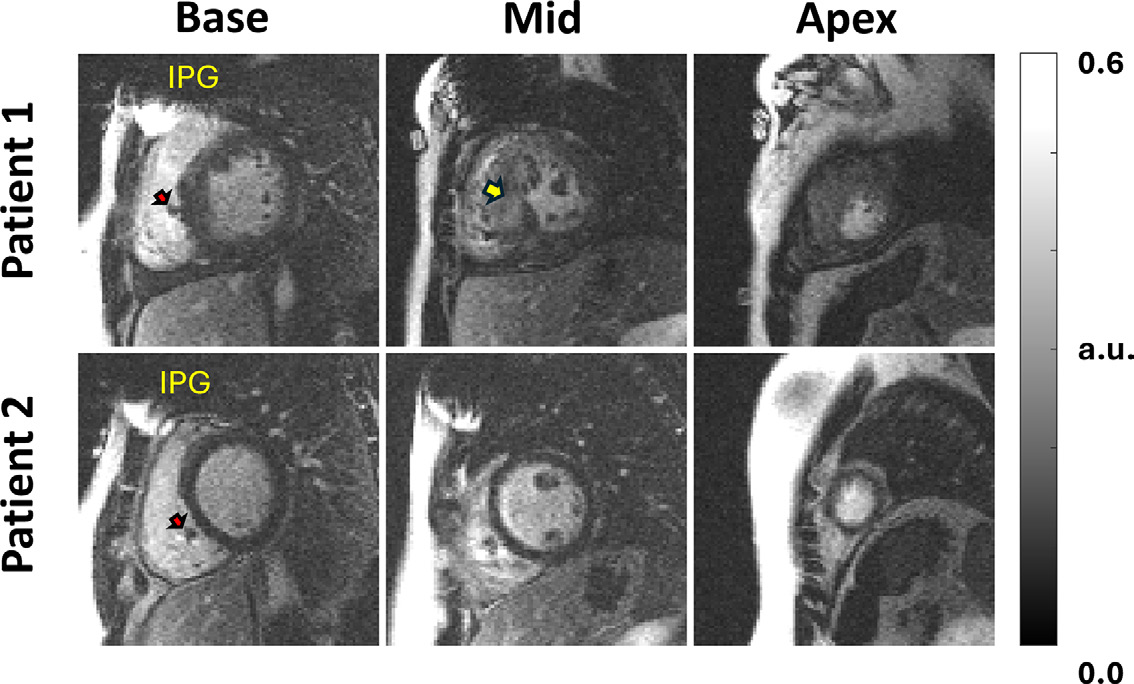
Wideband late gadolinium enhancement (LGE) images of two pediatric participants with cardiac implantable electronic devices (CIEDs) in situ, displaying basal, mid, and apical short-axis sections. The first participant (top row) with hypertrophic cardiomyopathy exhibited LGE patterns that are visible in the midventricular section (yellow arrow), whereas the second participant (bottom row) did not exhibit myocardial scarring. The red arrows point to signal void caused by the CIED lead. Participants are numbered in the order that they appear in Table 2. a.u. = arbitrary units, IPG = implantable pulse generator.

**Figure 6:**
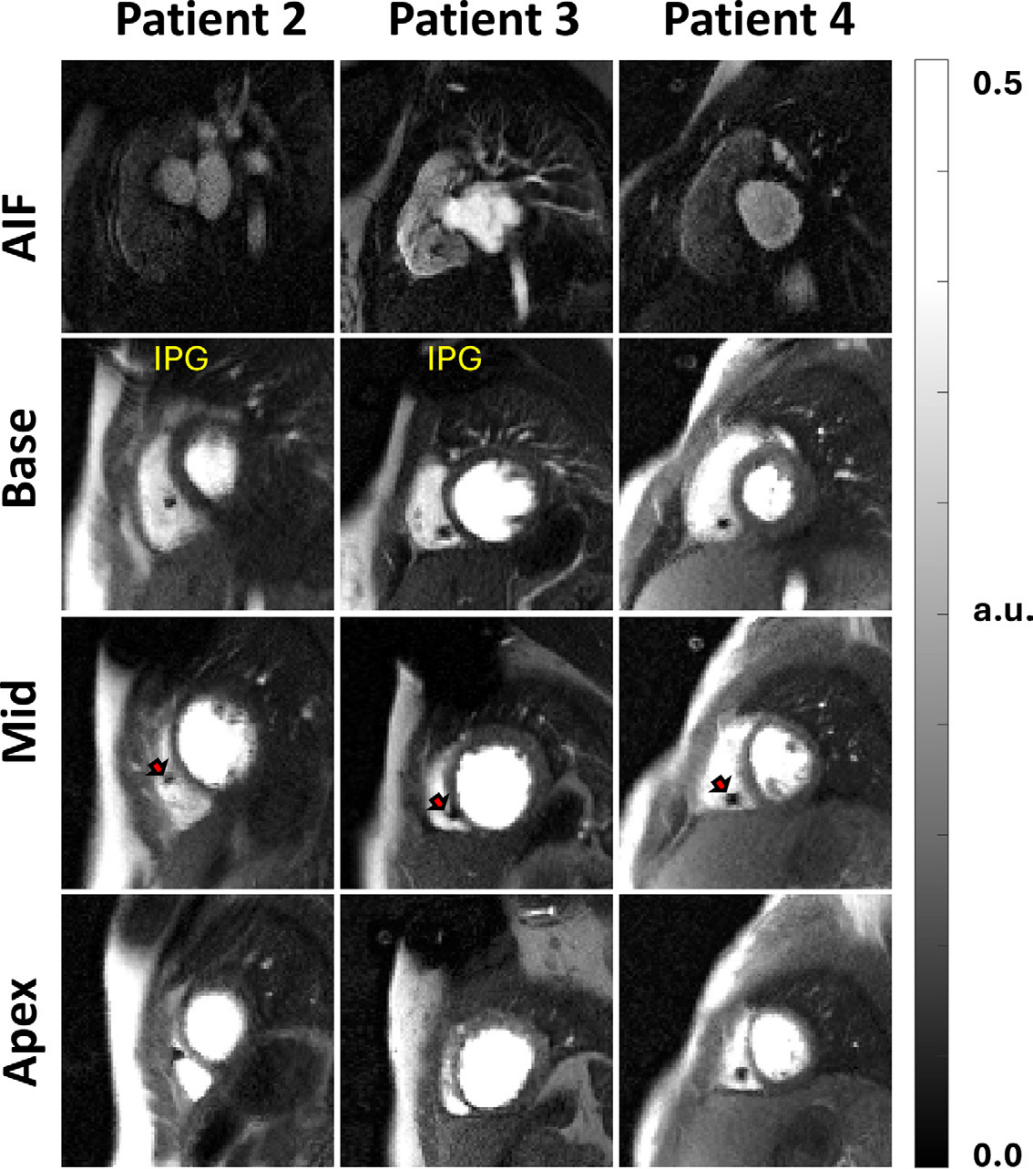
Wideband perfusion images of three pediatric participants with cardiac implantable electronic devices (CIEDs) in situ, showing the arterial input function image (top row) and the short-axis sections (base, mid, and apex). The red arrow points to the signal void caused by the CIED lead. Myocardial blood flow values were quantified on a pixel-by-pixel basis for each participant. Participants are numbered in the order in which they appear in Table 2. For dynamic display of the perfusion images, please see the Movie. AIF = arterial input function, a.u. = arbitrary units, IPG = implantable pulse generator.

Wideband LGE and perfusion scans were performed in four pediatric participants with CIEDs (median age, 15 years [range, 15–19 years]; three [75%] female) and an LV ejection fraction of 50.5% ± 16.4 (Table 1). Three participants had both LGE and perfusion scans, while one had only an LGE scan. Each participant had a median image quality score of 4.0 or more in all myocardial segments for both wideband LGE and perfusion images (Table 2). Similarly, all segments were clinically interpretable (no artifact) for both scans. Figures 5 and 6 show representative images for LGE and perfusion scans, respectively. Please see the Movie for a dynamic display of the perfusion images.

**Table 2:** Image Quality Scores Visually Assessed by Average Clinical Rater on Wideband LGE and Perfusion MRI of Pediatric Participants with CIEDs in Situ

Participants with CIEDs	Image Quality Score		Resting Myocardial Blood Flow (mL/min/g)
	LGE	Perfusion	
Participant 1	4.0 (3.0–4.0)	…	…
Participant 2	4.0 (3.7–4.0)	4.0 (3.7–4.0)	0.99 ± 0.30
Participant 3	4.0 (3.0–4.3)	4.0 (3.7–4.3)	1.19 ± 0.26
Participant 4	4.7 (4.7–4.7)	4.3 (3.7–4.3)	1.00 ± 0.30

Note.—Data are presented as medians with ranges in parentheses or means ± SDs (calculated from the perfusion images of each participant). The five-point Likert scale ranged from 1, nondiagnostic, to 5, excellent. CIED = cardiac implantable electronic device, LGE = late gadolinium enhancement.

**Movie:** Dynamic wideband perfusion images of three pediatric participants with CIED in situ, showing the arterial input function image (top row) and the short-axis sections (base, mid, and apex), corresponding to Figure 6 and Table 2 in the manuscript.

Table 2 summarizes the mean resting global myocardial blood flow values (range, 0.99 mL/min/g ± 0.30 to 1.19 mL/min/g ± 0.26) of participants with CIEDs scanned using the wideband perfusion sequence.

## Discussion

This study demonstrated the feasibility of wideband LGE and cardiac perfusion pulse sequences to suppress image artifacts induced by CIEDs in children at 1.5 T. The presence of an IPG taped in abdominal and subclavicular positions caused hyperintense artifacts in clinical standard LGE, whereas the corresponding wideband pulse sequences effectively suppressed image artifacts. Wideband LGE and perfusion pulse sequences produced high diagnostic image quality in pediatric participants with CIEDs in situ. Wideband perfusion sequence led to resting myocardial blood flow values (1.06 mL/min/g) that are consistent with literature values (0.94 mL/min/g) (18).

Our study in healthy pediatric participants with standard LGE and IPG taped to the subclavicular position showed that artifacts were greatest in the anterior LV myocardium, with severe artifacts in the base, mid, and apical segments. Although minimal artifact remained in the anterior segments with the wideband sequence, there was less artifact in each segment (Fig 2), and the posterior segments were nearly artifact free. Indeed, these results agree with previous studies in adult patients with CIEDs. For instance, Sasaki et al (3) and Rashid et al (5) showed that artifacts on standard LGE images in adult patients with left-sided endocardial implantable cardioverter defibrillator were localized to the anterior, lateral, and apical LV wall. In one study in adult patients, 50%–78% of the LV segments were deemed diagnostically interpretable with standard LGE, depending on the distance of the device to the heart (12). However, only 38% of the LV segments were clinically interpretable in our study in the pediatric participants in standard LGE with subclavicular IPGs. The difference in these findings could be due to the proximity of the IPG to the heart (3,12) and also highlights the impact of the size of the IPG relative to body size.

The Pediatric and Congenital Electrophysiology Society’s current guidelines recommend MRI scans in patients with epicardial or abandoned leads, based on individualized consideration of the risk-benefit ratio (19). The diagnostic yield and extent of imaging artifacts are directly relevant to the benefit analysis of that ratio. Notably, a recent multicenter review of children who had undergone cardiovascular MRI with a CIED in situ noted sufficient artifact to stop imaging in 3%–4% of scans but did not quantify the percentage of myocardial segments deemed clinically uninterpretable (20). Our results showed that epicardial CIEDs produced reduced image artifacts compared with endocardial CIEDs. Hospitals or imaging centers equipped with wideband cardiovascular MRI pulse sequences may be able to increase the benefit of cardiovascular MRI by suppressing image artifacts, with important and favorable implications for the risk-benefit calculation stipulated by the guideline.

This study had several limitations. First, the taped IPG study did not model image artifacts from CIED leads. As shown in our substudy of participants with CIEDs in situ, leads, which are composed of metal alloys, are expected to cause small signal voids around the leads (see Figs 5 and 6), but they do not produce hyperintense image artifacts as an IPG—composed of the titanium casing, battery, and electronics—would. Second, the risks of administering gadolinium-based contrast material to healthy children were considered unacceptable in this prospective participant study. However, unenhanced perfusion and LGE results were validated in participants with CIEDs who received gadolinium-based contrast material. Third, the sample size was small. A future study with a larger sample size with both endocardial and epicardial CIED systems is warranted. Fourth, we did not compare standard and wideband pulse sequences because our hospital’s MRI policy mandates the shortest possible protocols for pediatric patients with CIEDs to minimize risks. Fifth, although wideband radiofrequency pulses generate higher specific absorption rate than standard radiofrequency pulses, their duty cycle in perfusion (three times per heartbeat) and LGE (once every two heartbeats) is sufficiently low to remain within the Food and Drug Administration–approved specific absorption rate limit of 2.0 W/kg.

In pediatric participants with simulated IPGs and CIEDs in situ, wideband LGE and perfusion sequences can effectively suppress image artifacts, potentially enhancing cardiovascular MRI diagnostic accuracy in children with CIEDs.

## Supplemental Files

Appendices S1-S3, Table S1

Conflicts of Interest
