# Supplementary material for: Evaluation of Wideband Pulse Sequences for Suppressing Image Artifacts in Children with Cardiac Implantable Electronic Devices
Source: Radiol Cardiothorac Imaging. 2025 Nov 6;7(6):e250310. doi: 10.1148/ryct.250310 (PMC12728510; doi:10.1148/ryct.250310)
Supplement: Appendices S1-S3, Table S1 [file ryct250310suppa1.pdf]

Supplemental materials undergo peer review but are not copyedited, and may include typographical errors.

©RSNA, 2025
10.1148/ryct.250310

Appendix S1

MR Imaging and Pulse Sequence

A. LGE

All pulse sequences used a spoiled gradient-recalled echo readout to minimize off-resonance effects. The LGE scans were performed with phase-sensitive inversion recovery (PSIR) reconstruction (15). Approximately 10-12 short-axis (SAX) slices were scanned to cover the whole heart. For wideband LGE, a scout LGE was acquired in one SAX slice to potentially shift the center frequency of the wideband inversion pulse as needed. For standard and wideband LGE in the healthy pediatric volunteers, a uniform suppression of the myocardial signal is desired; therefore, the inversion time (TI) was set to 600 ms to null the native myocardium. The standard and wideband inversion pulse has a bandwidth of 1 kHz and 4 kHz, respectively.

B. Perfusion

We performed the perfusion scans in the basal, mid, and apical SAX slices. In each slice, both proton density (PD) and T1-weighted (T1w) images were acquired to calculate the normalized signal that corrects for coil profiles and unknown equilibrium magnetization. T1w images were acquired after a saturation time of 100 msec. The wideband SR pulse has a frequency bandwidth of 9 kHz compared to that of a standard SR pulse (2.5 kHz). As previously reported, the whole-body specific absorption rates of the wideband inversion and saturation pulses are well below 2 W/kg (7,12). Please see Table S1 for details on imaging parameters.

Supplemental materials undergo peer review but are not copyedited, and may include typographical errors.

Appendix S2

Quantitative Image Artifact Analysis

To quantify artifacts in the LGE images, we selected the three SAX planes (base, mid, apex) in the PSIR images, manually segmented the LV myocardium with in-house tools written in MATLAB (R2024a, Massachusetts, USA), and divided them into 16 segments. We calculated the mean signal intensity (SI) in each segment.

For perfusion, we normalized the T1w images with the PD images in the three SAX planes. We manually segmented the whole heart (both LV and RV), tracing the epicardial contour, since there is no contrast between blood and myocardium. Then we calculated the normalized SI (i.e., T1w/PD) (21). For both analyses, the standard and wideband scans with no taped IPG were used as references for their respective scan types. We specifically excluded voxels with signal voids to avoid spurious values arising from division by zero during image normalization.

Appendix S3

MBF Quantification

We quantified MBF (mL/min/g) in the wideband perfusion scans in MATLAB. As part of our workflow, the images were motion-corrected using NIH software (22). The signal-to-gadolinium concentration [Gd] conversion included the following steps: (a) The T1w images were normalized with PD images, (b) we derived the AIF signal-time curve by manually drawing a region of interest in the blood pool of the LV cavity, (c) Bloch equation was used to calculate T1 values from the signal (23), followed by a T2* decay correction (24) on the AIF signal-time curve before conversion to [Gd]-time curve, (d) we assumed fast water exchange (25) and Fermi as the transfer function (26) to calculate MBF on a pixel-by-pixel basis in the normalized T1w images of each three SAX

Supplemental materials undergo peer review but are not copyedited, and may include typographical errors.

slices. We calculated the mean rest MBF value for each patient by averaging the MBF value of each SAX slice.

Table S1: Summary of imaging parameters of standard and wideband perfusion and LGE pulse sequences.

	**Perfusion**		**LGE**	
**Parameters**	**Standard**	**Wideband**	**Standard**	**Wideband**
FOV (mm^2)	244-279 x 300-340	300-340 x 300-340	279 x 339	272 x 340
Slice Thickness (mm)	8	8	6	6
Acquisition Matrix	126 x 192	192 x 192	129 x 192	126 x 192
TR/TE (ms)	2.50 / 1.32	2.80 / 1.46	3.00 / 1.18	3.00 / 1.38
Flip Angle (°) – PD<br>Flip Angle (°) – T1w	5<br>15	5<br>15	-<br>15	-<br>15
TS /TI (ms)	100	100	600	590
Bandwidth (Hz/pixel)	1000	745	1000	1240
Scan Duration	20 heartbeats	20 heartbeats	2 heartbeats/slice	2 heartbeats/slice

†FOV: field of view, PD: proton density, TE: echo time, T1w: T1 weighted, TR: Repetition time, TS: saturation recovery time, TI: Inversion recovery time. There is no difference in the scan duration between standard and wideband sequences.

Supplemental materials undergo peer review but are not copyedited, and may include typographical errors.

References

21. Kim D, Oesingmann N, McGorty K. Hybrid adiabatic-rectangular pulse train for effective saturation of magnetization within the whole heart at 3 T. Magn Reson Med 2009;62(6):1368-1378. doi: 10.1002/mrm.22140

22. Benovoy M, Jacobs M, Cheriet F, Dahdah N, Arai AE, Hsu LY. Robust universal nonrigid motion correction framework for first-pass cardiac MR perfusion imaging. J Magn Reson Imaging 2017;46(4):1060-1072. doi: 10.1002/jmri.25659

23. Cernicanu A, Axel L. Theory-based signal calibration with single-point T1 measurements for first-pass quantitative perfusion MRI studies. Acad Radiol 2006;13(6):686-693. doi: 10.1016/j.acra.2006.02.040

24. Fan L, Allen BD, Culver AE, Hsu LY, Hong K, Benefield BC, Carr JC, Lee DC, Kim D. A theoretical framework for retrospective T2∗ correction to the arterial input function in quantitative myocardial perfusion MRI. Magn Reson Med 2021;86(2):1137-1144. doi: 10.1002/mrm.28760

25. Donahue KM, Weisskoff RM, Burstein D. Water diffusion and exchange as they influence contrast enhancement. J Magn Reson Imaging 1997;7(1):102-110. doi: 10.1002/jmri.1880070114

26. Jerosch-Herold M, Wilke N, Stillman AE. Magnetic resonance quantification of the myocardial perfusion reserve with a Fermi function model for constrained deconvolution. Med Phys 1998;25(1):73-84. doi: 10.1118/1.598163
